# The complete mitochondrial genome and phylogenetic analysis of *Batillaria cumingi* (Gastropoda: Batillariidae)

**DOI:** 10.1080/23802359.2020.1772685

**Published:** 2020-06-08

**Authors:** Chengrui Yan, Jiantong Feng, Yingying Ye, Jiji Li, Baoying Guo

**Affiliations:** a National Engineering Research Center for Marine Aquaculture, Zhejiang Ocean University, Zhoushan, China; b Key Laboratory of Informatization of Habitat Monitoring and Fishery Resource Conservation Research in the East China Sea of Zhejiang Province, Zhejiang Ocean University, Zhoushan, China

**Keywords:** *Batillaria cumingi*, mitochondria genome, phylogenetic, Illumina

## Abstract

We determined the complete mitochondrial genome of *Batillaria cumingi.* The *B. cumingi* mitochondrial genome is 16,100 bp in length, comprising 13 protein-coding genes, 22 transfer RNA genes, and two ribosomal RNA genes. The nucleotide composition for *B. cumingi* is 17.5% of C, 16.88% of G, 35.3% of T, and 30.31% of A. In 13 protein-coding genes, all genes start with ATG. For the stop codon, the cox2 gene stops with TTC, the cytb, nad1, and nad2 genes stop with TAG, and the other nine genes are with TAA. Of these 37 genes identified, nine protein-coding genes and six transfer RNA genes are encoded on the heavy strand and the other genes on the light strand. The phylogenetic tree was constructed based on 13 protein-coding genes of the *B. cumingi* and other 19 Gastropoda species, *Sepia latimanus* as outgroup using the Neighbour-joining method. The tree showed that the *B. cumingi* is closely related to the Semisulcospira coreana in Cerithioidea. We believe that this result will be helpful for the study of population genetic and phylogenetic analysis of the family Batillariidae.

*Batillaria cumingi* (Crosse, 1862) belongs to the family Batillariidaeis an intertidal gastropod. It is a kind of broad-temperate benthic gastropod which distributed in Japan, Korea and northern China (Okutani and Habe 1983). The shell of *B. cumingi* is cone-shaped and strong and the shell surface has low and thin longitudinal ribs. The species is algophagous, which lives on the mud beach in the middle and upper part of the intertidal zone, where wave intensity is low (Adachi and Wada 1997). At present, there is no research on the mitochondrial genome of *B. cumingi*. In this study, it is the first report of a complete mitochondrial genome sequence of *B. cumingi*. The specimen of *B. cumingi* was collected from Haikou, Hainan province, China (110.35°E, 20.02°N) and identified by morphology and deposited in Zhejiang Ocean University. The genomic DNA extraction was utilized the salting-out method (Aljanabi and Martinez 1997) with the muscle, then stored at −20 °C refrigerator in the National Engineering Research Center for Marine Aquaculture, Zhejiang Ocean University (specimen Accession number: BC20181001). The genomic DNA was prepared in 400 bp paired-end libraries, and The Illumina HiSeq X Ten platform was using total genomic DNA to sequence the mitochondrial genome. All the data were available and enumerated to the Microsoft oneDrive database (https://1drv.ms/w/s!ArF1Al5lLW_VatOzZ4ygq_H6jmY?e=Z3OYmF).

The *B. cumingi* mitochondrial genome is 16,100 bp in length (GenBank accession number: MT323103), comprising 13 protein-coding genes, 22 transfer RNA genes, and two ribosomal RNA genes. The nucleotide composition for *B. cumingi* is 17.5% of C, 16.88% of G, 35.3% of T, and 30.31% of A. In 13 protein-coding genes, all genes start with ATG. For the stop codon, the cox2 gene stops with TTC, the cytb, nad1, and nad2 genes stop with TAG, and the other nine genes are with TAA. Of these 37 genes identified, nine protein-coding genes and six transfer RNA genes are encoded on the heavy strand and the other genes on the light strand. The 12S rRNA is between the tRNA^Thr^ and tRNA^Ser^, and the 16S rRNA is between the tRNA^Val^ and tRNA^Leu^.

The phylogenetic tree was constructed based on 13 protein-coding genes of the *B. cumingi* and other 19 Gastropoda species, *Sepia latimanus* as outgroup using the Neighbour-joining method (Saitou and Nei 1987) by the program Phylip (Felsenstein 1989). The tree showed that the *B. cumingi* is closely related to the *Semisulcospira coreana* in Cerithioidea, similar to Cypraeidae and Architaenioglossa (Figure 1). We believe that this result will be one supplement of the genome information in mitochondrial of the family Batillariidae and facilitate the study on population genetic.

**Figure 1.**
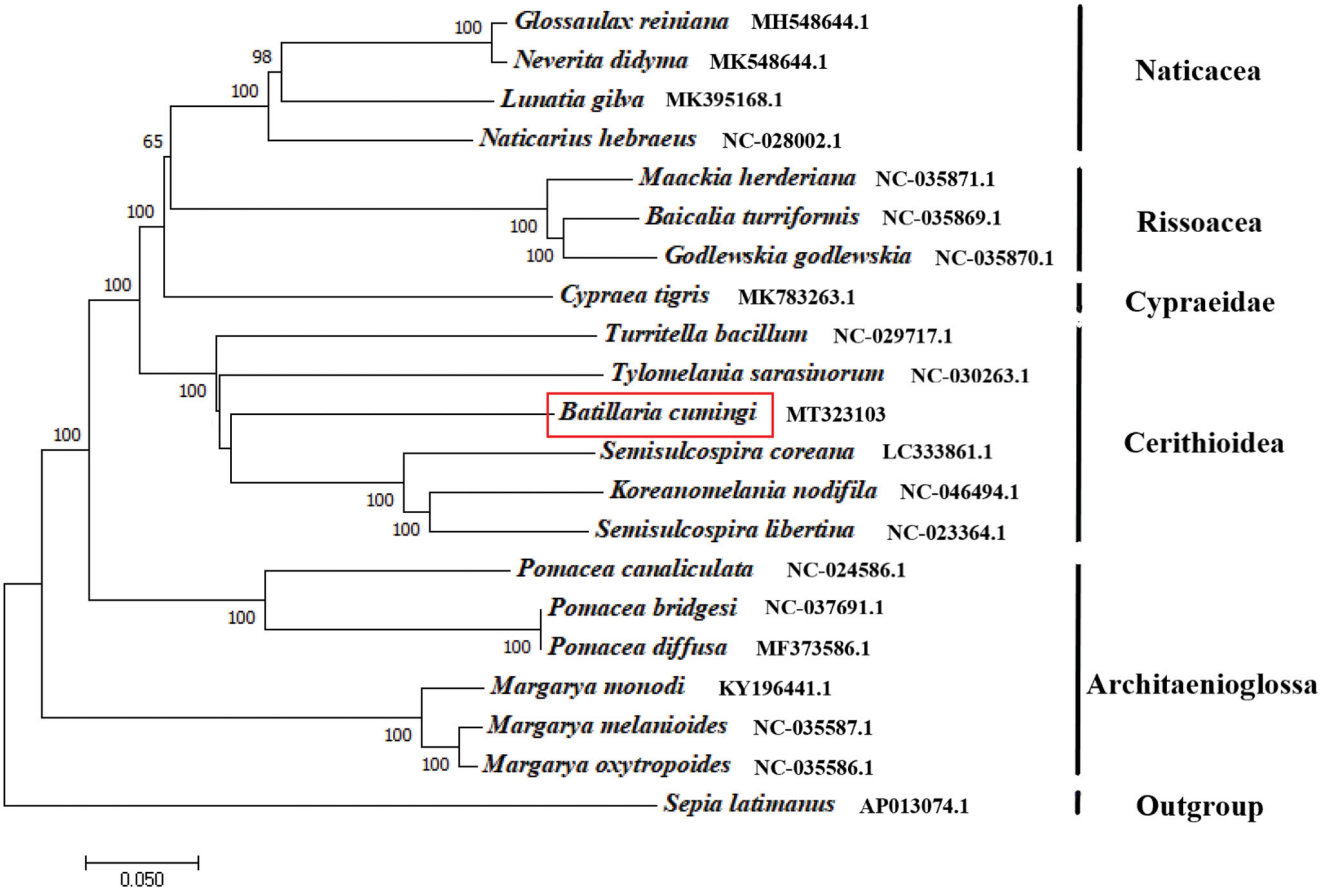
The NJ phylogenetic tree for *Batillaria cumingi* and other species based on 13 protein-coding genes.

## Data Availability

The data that support the findings of this study are openly available in Microsoft OneDrive at https://1drv.ms/w/s!ArF1Al5lLW_VatOzZ4ygq_H6jmY?e=Z3OYmF; and in Genbank, reference number: MT323103.
